# AID induces intraclonal diversity and genomic damage in CD86^+^ chronic lymphocytic leukemia cells

**DOI:** 10.1002/eji.201344421

**Published:** 2014-10-18

**Authors:** Michael Huemer, Stefan Rebhandl, Nadja Zaborsky, Franz J Gassner, Stefan Hainzl, Lukas Weiss, Daniel Hebenstreit, Richard Greil, Roland Geisberger

**Affiliations:** 1 Laboratory for Immunological and Molecular Cancer Research, Department of Internal Medicine III with Hematology, Medical Oncology, Hemostaseology, Infectious Diseases, Rheumatology, Oncologic Center, Paracelsus Medical UniversitySalzburg, Austria; 2 Division of Experimental Dermatology and EB House Austria, Department of Dermatology, Paracelsus Medical UniversitySalzburg, Austria; 3 School of Life Sciences, University of WarwickCoventry, UK

**Keywords:** Activation-induced deaminase (AID) • CD86 • Chronic lymphocytic leukemia (CLL) • Clonal evolution • Mutation

## Abstract

The activation-induced cytidine deaminase (AID) mediates somatic hypermutation and class switch recombination of the Ig genes by directly deaminating cytosines to uracils. As AID causes a substantial amount of off-target mutations, its activity has been associated with lymphomagenesis and clonal evolution of B-cell malignancies. Although it has been shown that AID is expressed in B-cell chronic lymphocytic leukemia (CLL), a clear analysis of in vivo AID activity in this B-cell malignancy remained elusive. In this study performed on primary human CLL samples, we report that, despite the presence of a dominant VDJ heavy chain region, a substantial intraclonal diversity was observed at VDJ as well as at IgM switch regions (Sμ), showing ongoing AID activity in vivo during disease progression. This AID-mediated heterogeneity was higher in CLL subclones expressing CD86, which we identified as the proliferative CLL fraction. Finally, CD86 expression correlated with shortened time to first treatment and increased γ-H2AX focus formation. Our data demonstrate that AID is active in CLL in vivo and thus, AID likely contributes to clonal evolution of CLL.

## Introduction

Chronic lymphocytic leukemia (CLL) is a malignant disease characterized by the accumulation of clonal CD5^+^CD19^+^ B-cells in the peripheral blood and lymphoid organs [1]. CLL is among the most common leukemias affecting primarily elderly patients and exhibiting an extremely variable clinical course. One of the strongest prognostic markers for CLL is the mutational status of the immunoglobulin variable (IgV) heavy chain at the rearranged VDJ region constituting the BCR, dividing the disease into IgV-mutated (IgV-Mut) and unmutated (IgV-UM) CLL, with IgV-UM having the worse prognosis [2],[3]. The activation-induced cytidine deaminase (AID) is a DNA-mutating enzyme responsible for somatic hypermutation and class switch recombination in normal germinal center B cells [4]. It initiates these processes by mutating cytosines to uracils within IgV and Ig switch (S) regions of the genomic DNA [5],[6]. As AID can also mutate off-target genes and aberrant AID-induced class switch recombination can lead to DNA double-strand breaks and genomic translocations, AID has been attributed a fundamental role in the development of germinal center-derived B-cell lymphomas [7]. Although AID mRNA was detected in CLL cells circulating in the peripheral blood [8]–[10], AID protein was only detectable in proliferating CLL cells which reside in lymph nodes or upon in vitro stimulation of peripheral CLL cells which are normally cell cycle arrested [11],[12]. This supports the hypothesis that CLL cells proliferate in germinal center-like proliferation centers in lymphoid tissue [13], where they upregulate AID upon interaction with T cells and other accessory cells. In line with this, CD86, a distinctive surface marker upregulated in germinal center light zone B cells [14], is also increasingly expressed on CLL cells residing in lymph nodes [15]. Surprisingly, AID expression was found to be higher in IgV-UM CLL cases and to correlate with a shorter treatment free and overall survival, irrespective of IgV mutation status, and with adverse risk cytogenetic aberrations [11],[12]. Conversely, although in vitro stimulated CLL cells show AID-dependent diversification of their IgV genes, IgV heterogeneity of CLL cells in vivo is low and restricted to a small subset of patients [12],[16].

From the current data it is hard to deduce whether AID implicates on CLL development by single mutagenic off-target events contributing to initial malignant transformation or whether AID implicates on clonal evolution of CLL by constantly mediating off-target DNA damage during disease progression. To draw light on these questions, we investigated mutational heterogeneity at the IgV as well as IgM switch regions (Sμ) which are the two main DNA regions subjected to AID-dependent mutations [6]. Ongoing AID activity during disease progression would result in a diverse intraclonal IgV pattern and in accumulation of “passenger” mutations at Sμ regions, thereby leading to IgV and Sμ heterogeneity. In addition, we sought to determine whether any IgV region heterogeneity could be discerned in a proliferative CLL subset, which would allow further conclusions on AID activity during disease progression.

## Results

### AID intraclonally diversifies IgV and Sμ regions of CLL cells in vivo

**Figure 1:**
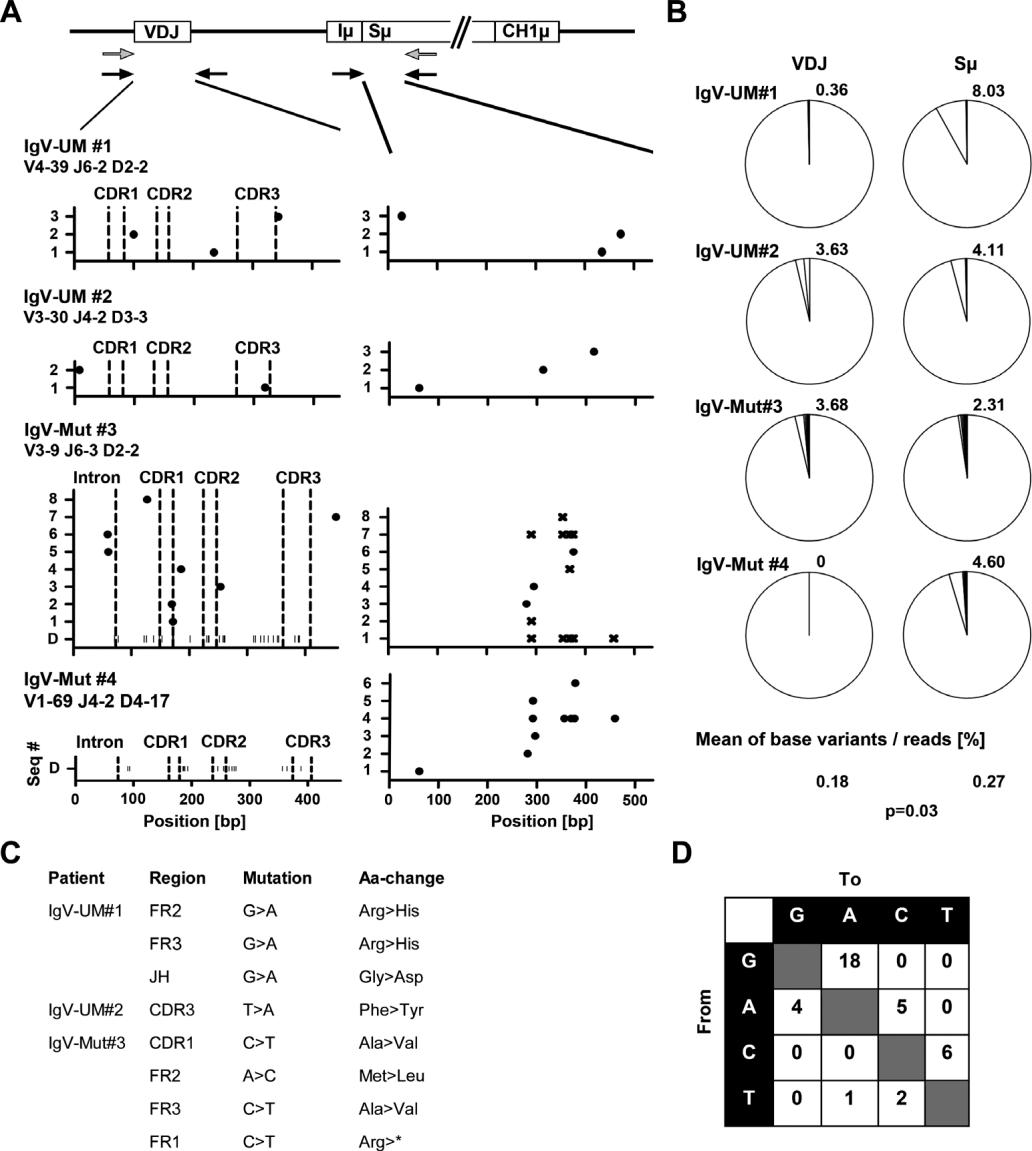
Sequence analysis of IgV and Sμ regions of two IgV-UM and two IgV-Mut CLL samples. (A) From four purified CLL samples, DNA was extracted and subjected to nested PCR to amplify and deep sequence the VDJ and Sμ regions from the rearranged allele. A schematic representation of the rearranged IgH locus is indicated, showing the VDJ gene, the Iμ exon, the Sμ region, and the first constant exon of the IgM heavy chain (CH1μ). Primer-binding sites are indicated (gray: first round PCR and black: second round PCR). The graphs show the set of subclonal VDJ and Sμ sequences (seq#) appearing within the individual samples with a frequency above 0.1%. The particular VDJ usage is indicated below each sample name. Dots within graphs display the position of bases that do not match with the dominant sequence. Base substitutions corresponding to germ line sequences in Sμ are indicated with x. IgV mutations of the dominant clone (D) are indicated as vertical bars along the *x*-axis for the two IgV-Mut samples. (All sequences are listed in Supporting Information Table 3.) (B) The frequency of subclonal sequences was determined by dividing the number of sublonal sequences gained by next generation sequencing (NGS) by the number of total sequences. The number above each pie chart gives the percentage of all subclonal variations of the respective sequence (only sequences with a frequency >0.1% were counted). The mean percentage of base variants/sequence for all IgV versus Sμ regions and the resulting *p*-value are indicated on the bottom of the graph. Data are compiled from one NGS experiment on four samples (#1–4). (C) Replacement mutations at VDJ genes of all subclonal variations shown in (A) were determined according to the genetic letter code. Nonsense mutations are marked with an asterisk. (D) The mutation spectrum for all VDJ and Sμ mutations depicted in (A) is shown. Nucleotides are listed on the axes and the numbers in each box represent the number of the respective mutation.

AID on-target mutations are restricted to genomic IgV and Sμ DNA regions. To investigate AID activity in CLL, we developed a nested PCR approach to amplify the genomic region spanning the rearranged VDJ gene downstream to the Sμ region using clone-specific V-gene primers and Sμ-specific consensus primers on genomic DNA from two IgV-UM (IgV-UM #1, 2) and two IgV-Mut samples (IgV-Mut #3, 4). For the second round of amplification, tagged primers were used to separately amplify the VDJ region and the 5′-Sμ region of the individual patient samples. The amplicons were pooled and deep-sequenced to obtain a full spectrum of mutations present in leukemic subsets of the CLL samples. To minimize PCR artifacts and sequencing errors, we only analyzed quality-filtered sequences (as described in Materials and methods) with a prevalence of >0.1% (listed in Supporting Information Table 3). In total, 842 862 quality-filtered sequences were analyzed for subclonal VDJ and Sμ region variants within the four selected CLL samples (a minimum of 12 786 sequences descending from one region per sample were analyzed). In line with earlier reports [17], we observed a slight intraclonal diversity at the VDJ gene in three of four samples reflected in the occurrence of two to eight additional subclones with a total prevalence of 0.36–3.68% (Fig.1A and B). Ten of the observed 13 mutations within VDJ genes were located in coding regions and eight of these affected the amino acid sequence (Fig.1C and Supporting Information Fig. 1). One C >T mutation resulted in a premature stop codon, however this mutation occurred with a borderline frequency of 0.11% and might be due to a PCR artifact as CLL cells strongly rely on the expression of a functional BCR for survival [18]. At the Sμ regions, we observed intraclonal diversity in all four samples with three to eight additional subclones appearing with a frequency of 2.31 to 8.03% of all counted reads (Fig.1A and B). Alignment with germline Sμ sequences revealed that the dominant clone harbored no mutations in Sμ with the exception of IgV-Mut #3, where the major Sμ clone exhibited five mutations. However, the second most frequent clone of this sample (0.61% frequency) appeared to be the unmutated Sμ region, implying that either these five mutations accumulated during disease progression or PCR-contamination with germline DNA occurred. In line with this, in contrast to VDJ regions, repeated mutations were observed at several positions in Sμ regions from the two IgV-Mut samples (Fig.1A, Supporting Information Table 3). Comparing the mutation frequency at IgV versus Sμ revealed that Sμ is mutated more efficiently than IgV (Fig.1B; 0.18 versus 0.27% mutations/sequence; *p* = 0.03 Mann–Whitney test). All subclonal mutations revealed a high proportion of C > T/G > A transitions (24 of 36 mutations; Fig.1D), which, although atypical for somatic hypermutation where transition to transversion ratios are ≈1:1, points to the action of cytidine deamination.

### CD86 expression defines proliferative CLL cells with a distinctive intraclonal IgV region diversity

**Figure 2:**
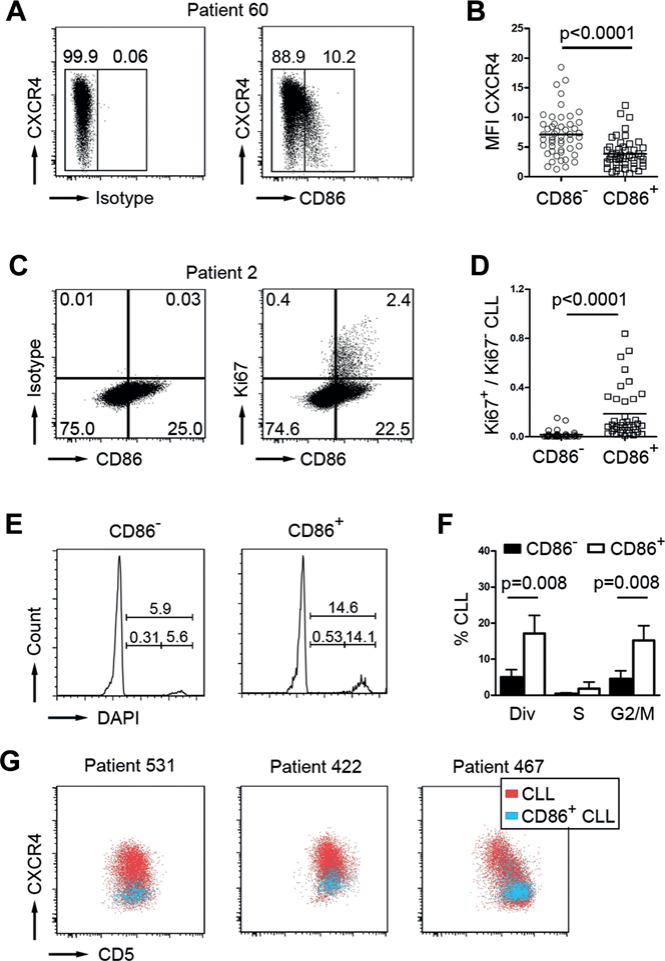
CD86^+^ cells represent the proliferative CLL fraction. (A) Representative flow cytometry profile of a CLL sample showing CXCR4 and CD86 expression on CD5^+^CD19^+^ pregated CLL cells. Boxes and numbers in dot plots indicate gating for CD86 positive versus negative cells. Plots are representative of 50 independent experiments. (B) MFI values of CXCR4^+^ cells are quantified for CD86^–^ and CD86^+^ CLL cells (pregated for CD5^+^CD19^+^ expression; each individual patient sample is represented by a single circle symbol (CD86^−^ fraction) and quadrant symbol (CD86^+^ fraction), *n* = 50; mean values shown as horizontal lines; *p*-value determined by paired *t*-test). (C) Representative flow cytometry profile for CD86/Ki67 coexpression on CD5^+^CD19^+^ pregated CLL cells. Boxes and numbers in dot plots indicate percentages of cells within the respective quadrants. Plots are representative of 38 independent experiments. (D) The ratio of Ki67^+^/Ki67^−^ CLL cells within CD86^−^ and CD86^+^ subsets was determined by flow cytometry based on the quadrant percentages shown in (C). Each individual patient sample is represented by a single circle symbol (CD86^−^ fraction) and quadrant symbol (CD86^+^ fraction), *n* = 38; mean values shown as horizontal lines; *p*-value determined by Mann–Whitney test. (E) Representative cell cycle profiles of CD86^−^ and CD86^+^ CLL fractions (pregated on CD5^+^CD19^+^ cells) of patient ID 565, determined by DAPI DNA staining. Percentages are shown within the FACS graphs. Histograms are representative of five independent experiments. (F) Cell cycle profiles were analyzed by plotting the percentages of CLL cells within the respective cell cycle phase as determined in (E) for CD86^−^ (black bars) and CD86^+^ (white bars) CLL cells; (div = dividing cells in M/S/G2; *n* = 5; bars show means ± SD; *p*-values determined by Mann–Whitney test). (G) Flow cytometry was used to examine the expression pattern of CD5 and CXCR4 on CD86^+^ CD5^+^CD19^+^ CLL cells (blue) within the total population of CD5^+^CD19^+^ CLL cells (red). Dot plots are representative of 50 independent experiments.

**Figure 3:**
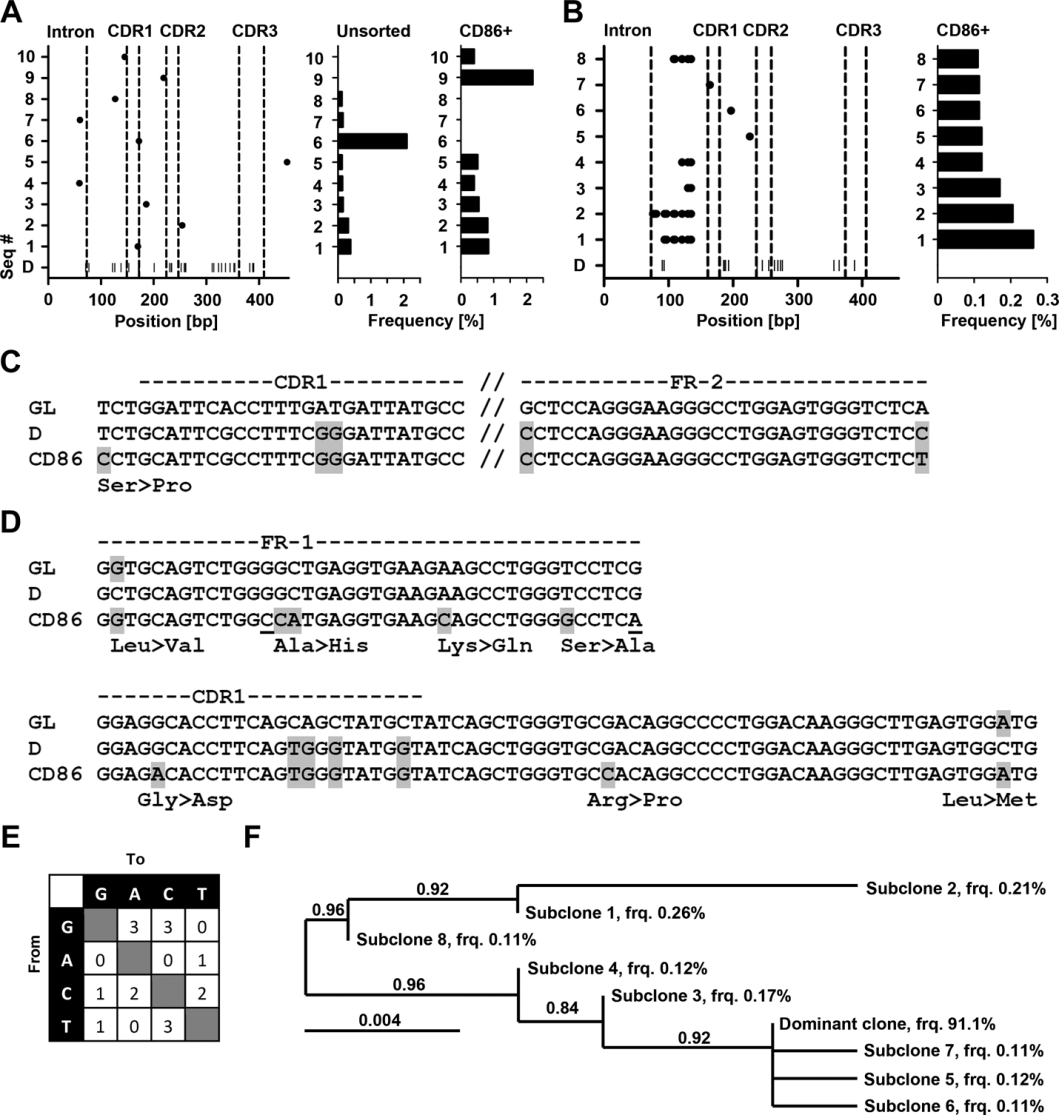
IgV and Sμ region mutations in unsorted versus CD86^+^-sorted CLL cells. The diverse set of VDJ sequences (seq#) for (A) IgV-Mut #3 and (B) IgV-Mut #4, appearing with a frequency >0.1%, is shown for unsorted and CD86^+^-sorted CLL cells as described in Figure1. (For IgV-Mut #4, only sequences from CD86^+^-sorted samples are shown as no subclonal variations were detected in unsorted samples.) The relative frequencies of the subclones are indicated as horizontal bars on the right of each graph. Data are compiled from one NGS experiment on pooled tagged amplicons derived from DNA of >50 000 sorted cells. Sequences of subclonal VDJ mutations from CD86^+^-sorted samples of (C) IgV-Mut #3 and (D) IgV-Mut #4 are shown in alignment with germ line V sequences (GL) and the respective dominant clone (D). Amino acid changes are shown underneath the alignment and are indicated in gray. Silent mutations are underlined. The resulting mutation spectrum is shown in (E). Nucleotides are listed on the axes and the numbers in each box represent the number of the respective mutation. (F) Genealogical relation of VDJ mutations from CD86^+^-sorted IgV-Mut #4 is given. The frequency (frq) of the individual subclones is indicated. The branch support values are given within the diagram and the length of each branch is proportional to the number of varying bases (evolutionary distance).

**Figure 4:**
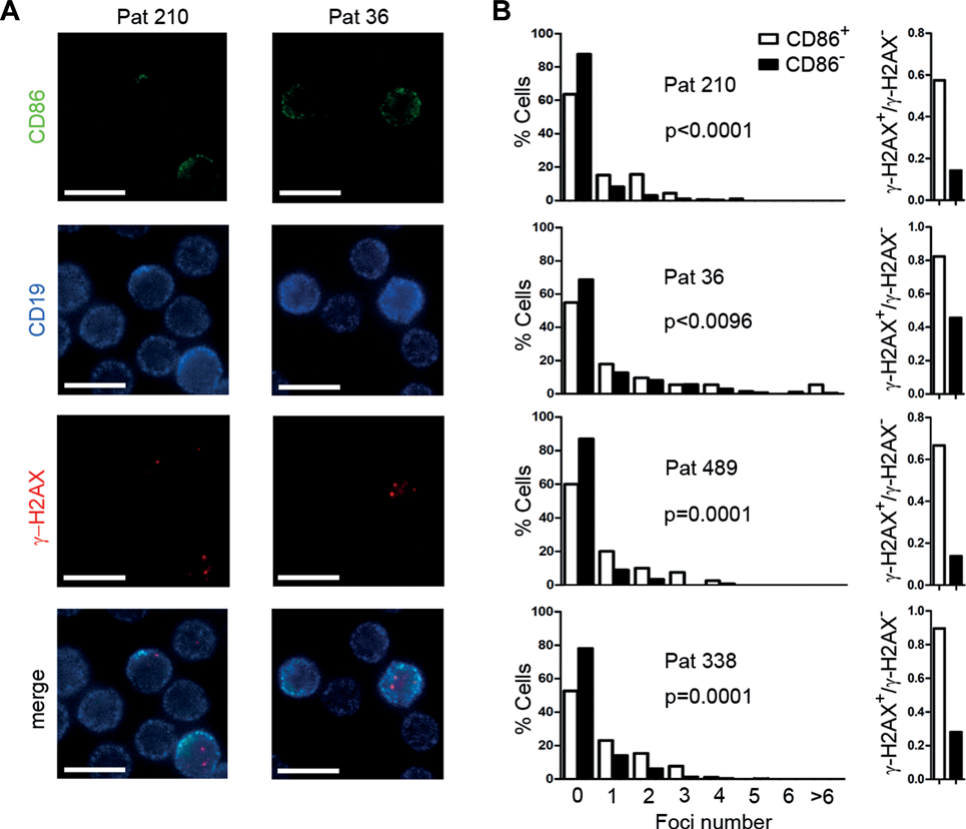
CD86^+^ CLL cells harbor more y-H2AX foci. CLL cells were stained for CD19, CD86, and γ-H2AX, and assessed by fluorescence microscopy. The number of γ-H2AX foci was counted in CD86^+^ and CD86^−^ CLL cells. (A) Micrographs of two patients stained for CD86, CD19, and γ-H2AX. Bars, 10 μm. Images are representative of four independent experiments. (B) Results obtained as in (A) are depicted as the percentage of cells harboring the indicated foci number (left panel), and as fractions of γ-H2AX^+^/γ-H2AX^−^ cells within CD86^+^ (white bars) and CD86^−^ (black bars) CLL cells (right panel). Data were generated by analyzing the following number of cells by fluorescence microscopy as shown in (A): ID 210 *n* = 1126; ID 36 *n* = 1133; ID 489 *n* = 159; ID 338 *n* = 941. *p*-Values indicate significance of the difference in mean foci number between CD86^+^ versus CD86^−^ CLL cells, determined by Mann–Whitney test.

Since we observed subclonal heterogeneity in CLL, we asked whether we could discern a subset of CLL cells in which novel AID-dependent mutations accumulate. As AID was reported to be upregulated in proliferating CLL cells in lymph nodes [11],[12], we suspected that intraclonal diversity at IgV and Sμ regions would reflect the degree of iterative cycling of CLL cells through germinal center-like lymphoid structures. Normally, germinal center B cells are divided into light zone and dark zone cells which are characterized by their distinct expression of activation marker CD86 and chemokine receptor CXCR4 with light zone cells being CD86^hi^CXCR4^lo^ and dark zone cells CD86^lo^CXCR4^hi^ [14],[19]. Light zone cells either return to the dark zone for further rounds of proliferation, or eventually exit from the germinal center to plasma/memory cell fate [20]. Based on the assumption that CLL cells that recently emigrated from lymphoid niches would retain a light zone specific surface profile, we tested whether CD86^hi^CXCR4^lo^ cells could be discerned in the peripheral blood of CLL patients. Indeed, we found that a fraction of CLL cells was CD86^hi^CXCR4^lo^ (Fig.2A and B). Cell cycle analysis revealed that peripheral CD86^hi^CXCR4^lo^ CLL cells were more frequent in G2 and M phase than their CD86^lo^ counterparts (Fig.2E and F). In line with that, expression of the proliferation associated antigen Ki67 was higher in CD86^hi^CXCR4^lo^ CLL cells than in CD86^lo^ CLL cells (Fig. 2C and D). Moreover, CD86^hi^CXCR4^lo^ CLL cells overlapped with the CXCR4^lo^CD5^hi^ CLL subset, which represents CLL cells previously described as a recently divided CLL fraction that emigrated from lymphoid tissue (Fig.2G) [21]. Assuming that CD86^+^ CLL cells represent a proliferative fraction which recently emigrated from lymphoid tissue, we suspected this fraction to harbor a higher intraclonal IgV region complexity. Thus, we sorted CD86^+^ CLL cells from two IgV-Mut CLL patients (IgV-Mut #3, 4) and examined the mutational diversity at the rearranged VDJ region by our deep-resequencing approach. We chose to analyze the VDJ region instead of the Sμ region as the use of V-specific primers precludes PCR amplification of contaminating DNA and hence, likely yields more reliable results. In both samples, we found that the same dominant VDJ clones appeared in unsorted and CD86^+^-sorted samples (Fig.3, Supporting Information Table 4). For IgV-Mut #3, several subclonal VDJ sequences were shared between sorted and unsorted cells (Fig.3A). Unique sequences were observed for CD86^+^-sorted cells from both samples, which exhibited additional mutations in VDJ regions which were beyond detection limit (<0.1%) in the unsorted samples (Fig.3A and B). For IgV-Mut #4, the CD86^+^-sorted subset harbored sequences with additional mutations which were genealogically related (Fig.3F). Two of these mutations showed the germ line sequence (Fig.3D). Either, these are genuine hypermutation events occurring at an RCY AID hotspot motif or otherwise our deep-sequencing approach revealed the presence of a common ancestor clone within the CD86^+^ fraction. The sequences from CD86^+^-sorted CLL cells showed a typical somatic hypermutation spectrum with an equal amount of transitions and transversions occurring (Fig.3E). Moreover, fluorescence microscopy revealed that CD86^+^ CLL cells showed an increase in γ-H2AX foci, a marker for double-strand DNA breaks [22]. The γ-H2AX foci number per cell as well as the amount of γ-H2AX positive cells were higher in the CD86^+^ subset of CLL cells (Fig.4), which is in line with an activated B-cell phenotype [23].

### CD86 expression correlates with treatment free survival

**Figure 5:**
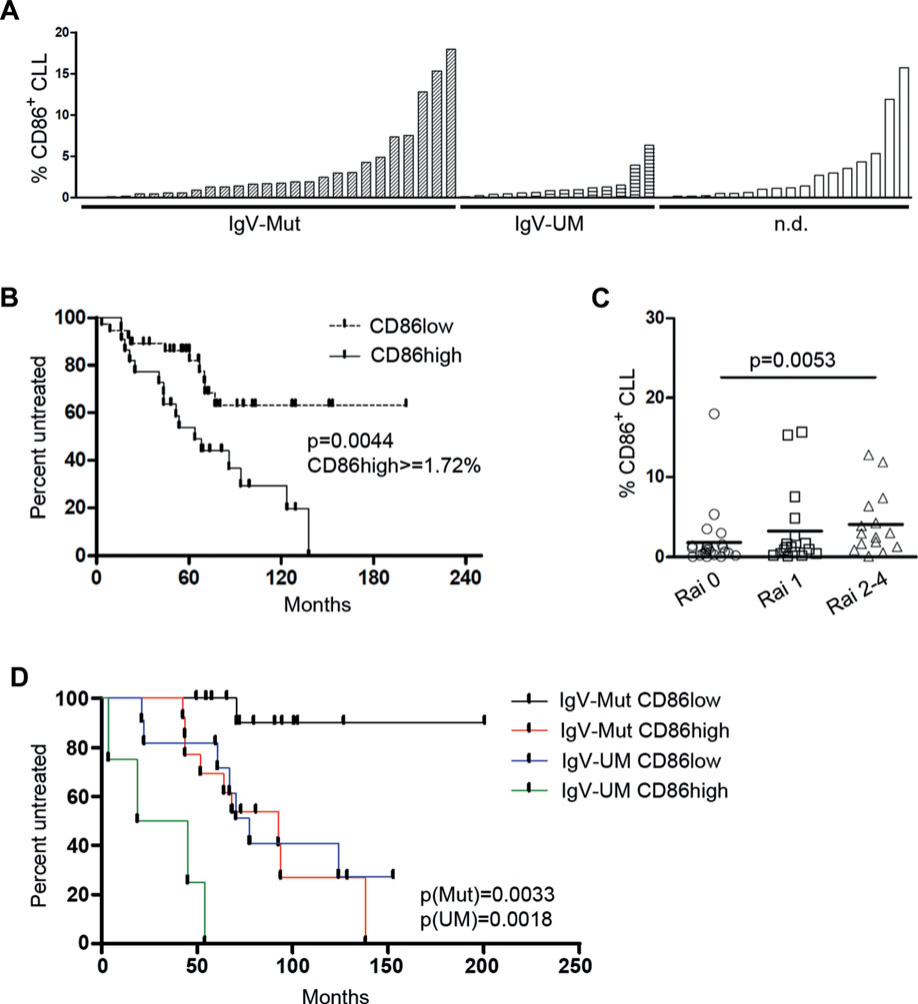
Increased CD86 expression associates with severe disease. (A) Percentages of CD86^+^ cells within a cohort of 59 previously untreated CLL patients were assessed by flow cytometry. Mutation status of the IgVH gene of each patient is indicated on the *x*-axis. n.d.: mutation status not defined. Each bar represents an individual patient sample analyzed for CD86^+^ CLL cells in one single experiment. (B) Time to first treatment was investigated in 59 CLL patients shown in (A) using Kaplan–Meier analysis. (*n* = 59). Symbols on each line indicate censored events. *p*-Values for comparison of risk groups were determined using the log-rank test. (C) Patients were stratified into three risk groups according to Rai staging (Rai 0, 1, and ≥2; *n* = 56). Each symbol represents an individual patient and the mean is shown with a horizontal line. *p*-Values were determined by Mann–Whitney test. (D) Time to first treatment was investigated in CLL patients shown in (A), using Kaplan–Meier analysis. (*n* = 59). Symbols on each line indicate censored events. Patient details are provided in Supporting Information Table 1.

To evaluate whether the presence of CD86^+^ CLL cells impacts on the course of disease, we measured CD86 levels in a set of 59 previously untreated CLL patients (Supporting Information Table 1) and correlated the size of the CD86 fraction in the peripheral blood with time to first treatment. Using a cut-off of ≥1.72% CD86^+^ CLL cells as determined by receiver operating characteristic analysis, we classified this patient cohort into two risk groups and performed a Kaplan–Meier analysis. The patient group with ≥1.72% CD86^+^ CLL cells (CD86^high^) had a significantly shorter time to first treatment than the CD86^low^ (<1.72% CD86^+^ CLL cells) subgroup (log-rank *p* = 0.0044; median time to first treatment for CD86^high^ = 63.7 months, for CD86^low^ = not reached; Fig.5B). Also within the IgV-UM and IgV-Mut CLL groups, patients could be classified into two risk groups with significant differences in time to first treatment (IgV-Mut: log-rank *p* = 0.0033; IgV-UM: log-rank *p* = 0.0018; Fig.5D). Moreover, in our patient cohort, the percentage of CD86^+^ CLL cells was higher in patients with advanced Rai stage (Fig.5C). Finally, the percentage of CD86-expressing CLL cells in the peripheral blood remained an independent prognostic marker together with the IgVH mutation status in a multivariate analysis (*p* = 0.001 and *p* = 0.001; Table1).

**Table 1:** Correlation of CLL risk factors and CD86 expression

	Cox regression proportional hazard					
	Univariate			Multivariate		
Parameter	*p*	HR	95% CI	*p*	HR	95% CI
IgVH	0.022	2.81	1.16–6.8	0.001	7.67	2.47–23.82
del17p; del11q	0.207	0.564	0.232–1.373	-	-	-
Zap70^+^	0.060	2.22	0.97–5.09	-	-	-
CD38^+^	0.086	1.98	0.91–4.32	-	-	-
CD86^+^	0.007	2.99	1.35–6.59	0.001	6.57	2.13–20.21

Cox regression analysis was performed to test association between each parameter and the treatment free survival of 59 CLL patients.

HR: hazard ratio; CI: confidence interval.

## Discussion

Acquisition of somatic mutations and chromosomal aberrations is a hallmark of cancer development and clonal evolution of cancer cells [24]. In CLL, only a few recurrent lesions were initially identified and associated with clinical prognosis, such as mutations of the tumor protein 53 gene (*TP53*) or chromosomal 17p13 deletions abrogating p53 expression [25],[26]. Also mutations of the ataxia telangiectasia mutated gene or a deletion of ataxia telangiectasia mutated gene encoding region del(11)(q22.3) has been associated with pathogenesis and have prognostic value [27]. However, cytogenetic analyses have indicated the presence of frequent complex chromosomal alteration in CLL [28] and recent advances in high throughput sequencing have revealed an even more complex genetic landscape, with novel mutations of prognostic power being identified such as mutations within the genes encoding Notch1, MyD88, Birc3, and Xpo1 [29],[30]. In addition to recurrent mutations in these four genes, approximately 1 000 mutations in nonrepetitive regions were detected per CLL case together with large genomic alterations [29]. Also massive remodeling of single chromosomes, termed chromothripsis, involving ten to hundreds of genomic rearrangements were detected in CLL cells and found to be associated with poor clinical outcome [31],[32]. The most frequent mutations detected by high throughput sequencing were C to T transitions, usually occurring in a CpG context [29]. C to T transitions normally occur upon deamination of Cs, leading to the generations of Us. Deamination of Cs can either occur by spontaneous hydrolysis or by the enzymatic activity of deaminases.

The DNA deaminase AID is a member of the APOBEC family of deaminases and was recently found to be expressed in CLL cells [33]. APOBEC3, another APOBEC family member was recently found to be implicated in initiating clustered off-target mutations in CLL [34]. As AID can deaminate unmethylated [5] as well as methylated Cs [35], it is conceivable that AID together with other APOBEC family members is implicated in the generation of clustered or unclustered C to T transitions in CLL. The physiological role of AID is to deaminate Cs within the Ig loci. The subsequent error prone repair of generated Us leads to somatic hypermutation in IgV regions and to double strand (ds) DNA breaks within S regions. The nonhomologous end joining of dsDNA breaks in different S regions finally leads to class switch recombination [6]. Considerable genome wide off-target deamination activity outside the Ig loci was reported for AID [36] and brought in context to germinal center-derived lymphoma development [37]. Although it is still unclear whether CLL is a germinal center-derived malignancy [38], several reports describe the presence of AID mRNA transcripts in peripheral blood CLL cells [10],[12],[39] and high AID levels were associated with Richter's transformation, where CLL transforms into a highly aggressive B-cell lymphoma [40]. AID protein was detectable in lymph node residing CLL cells [11], where interaction with accessory cells is thought to initiate CLL cell activation and proliferation. Consequently, activation of CLL cells in vitro by CD40L/IL-4 also leads to substantial upregulation of AID protein [12]. In those studies, AID levels were found to be associated with IgV-UM CLL, with chromosomal aberrations and a shorter overall survival as well as shorter time to first treatment [9],[10],[12]. Although these data point to a likely role of AID in disease pathogenesis in CLL, the evidence for in vivo AID activity is scarce: typical AID off-target damage, such as somatic mutations of the BCL6 gene [41] or c-myc/IgH translocations [42] are very rare events in CLL and IgVH genes in AID-expressing CLL cells are frequently unmutated. In addition, although CLL cells diversify their IgV genes upon in vitro stimulation, the intraclonal diversity at IgV regions in vivo is rather low compared to other AID-expressing B-cell malignancies, such as follicular lymphoma [43],[44].

To elucidate ongoing AID activity in CLL in vivo, we investigated mutational diversity of IgV and Sμ region DNA which both are bona fide AID targets. However, unlike IgV mutations which affect antigen-binding specificity, mutations at Sμ regions are “passenger” mutations as they do not encode proteins and thus are unselected tracers for AID activity. Although previous reports described the presence of mutated Sμ regions also in IgV-UM cases, the intraclonal diversity at Sμ regions, and thus indication for ongoing AID activity has not been assessed

We therefore developed a nested PCR-based deep-sequencing approach to validate intraclonal IgV and Sμ mutations in CLL samples. A challenge in this approach is certainly to distinguish between genuine mutations and artifacts due to PCR errors, library preparation, or sequencing and we therefore quality-filtered all sequences and only analyzed sequences with a frequency above 0.1%. This cut-off led to the occurrence of only one VDJ clone for sample IgV-Mut #3 and thus, was judged as adequate to eliminate artifacts even though genuine subclonal mutations might be missed. The subclonal mutations observed by this method likely arise through AID-mediated somatic hypermutation and thus give a profound evidence for AID activity during disease progression in IgV-Mut as well as in IgV-UM cases. Our analysis revealed that subclonal mutations are not limited to the VDJ locus but also the Sμ region is subjected to an even higher somatic hypermutation in CLL. Overall, the level of AID-induced mutations—though clearly detectable by our sequencing approach—was rather moderate as only a small percentage of all sequences consisted of subclonal variations. Of note, the dominant Sμ sequence in IgV-Mut #3 harbored five mutations even though the germ line Sμ sequence was still detectable within the subclones. From these data, it is tempting to speculate that the five Sμ mutations accumulated during disease progression from an Sμ-unmutated ancestor clone, however, as the Sμ primers, unlike the IgV primers, can also specifically bind to DNA from non-B cells, the analysis of Sμ mutations likely assumes a higher risk of PCR artifacts due to template contamination with DNA from non-B cells. Hence, we chose to investigate subclonal IgV mutations in all further experiments.

Finally, we revealed that a fraction of peripheral blood CLL cells express surface CD86 and by purifying this subpopulation from two patients we showed that they carried additional and partially clonally related IgV sequences. Based on these mutations, we propose that the CD86^+^ fraction in the peripheral blood comprises CLL cells that recently emigrated from lymphoid niches, where they diversified the Ig locus in an AID-dependent manner. In IgV-Mut #4, the novel mutations in the CD86^+^ fraction were all genealogically related and might reflect the consecutive accumulation of hypermutation events. Two of these mutations showed the germ line sequence, however they could well result from consecutive mutations as these bases are cytidines located within an RCY AID hotspot motif. Otherwise, a common ancestor clone could be involved in the accumulation of consecutive mutations in the CD86^+^ fraction. In addition, the CD86^+^ fraction showed a higher expression of the proliferation-associated antigen Ki67 and was to a higher extent in G2/M phase of the cell cycle. In line with this, the CD86^+^ population overlapped with CD5^hi^CXCR4^lo^ CLL cells which were reported to comprise recently divided cells based on their incorporation of heavy water in vivo [21]. Furthermore, CD86^+^ CLL cells exhibited a higher amount of γ-H2AX foci, which is indicative for a higher incidence of DNA damage. The appearance of γ-H2AX foci formation is generally coupled to B-cell activation and could be a direct result of AID-mediated DNA damage also outside the Ig loci [23]. Error prone repair of γ-H2AX-marked double-strand DNA breaks by nonhomologous end joining could therefore directly contribute to the clonal evolution in CLL [45]. Concomitantly, a recent report showed that CLL samples expressing AID transcripts harbored significantly more γ-H2AX foci than non-AID-expressing samples when dsDNA repair by homologous recombination is inhibited, indicating AID-induced DNA damage [46]. Beyond that, an augmented CD86^+^CXCR4^lo^ CLL subpopulation in peripheral blood was an independent predictive marker for shorter time to first treatment, which further supports our hypothesis that CD86 indicates the magnitude of CLL cells cycling through proliferation centers, and hence, is associated with intraclonal diversification. Notably, CD86^+^ CLL cells were predictive for time to treatment in both IgV-Mut as well as in IgV-UM cases, which corroborates our data from targeted VDJ/Sμ resequencing, showing that AID is active in IgV-Mut as well as IgV-UM samples. Thus, although higher transcript levels were reported for IgV-UM CLL, AID protein is active in CLL irrespective of the IgV-mutation status, thereby generating subclonal IgV/Sμ heterogeneity and likely mediating off-target DNA damage which contributes to clonal evolution.

In conclusion, our data show in vivo activity of AID in CLL samples as reflected by a diverse set of IgV and Sμ sequences and the accumulation of unique mutations in the CD86^+^ CLL subset. This CD86 positive CLL subset represented the proliferating CLL fraction, accumulated dsDNA breaks, and predicted for shorter treatment free survival.

## Materials and methods

### CLL samples

Peripheral blood samples from CLL patients from our outpatient care facility at the IIIrd Medical Department were collected upon informed consent in accordance with the Declaration of Helsinki and upon approval by the ethics committee of Salzburg, Austria (ref. no. 415-E/1287/8–2011). For time to treatment analysis, samples from 59 previously untreated patients (Supporting Information Table 1) were collected or thawed from our biobank after confirming that freezing and thawing of samples does not influence CD86 expression values (Supporting Information Fig. 2). At the time of study, median time from diagnosis was 48 months. The assessment of immunoglobulin heavy chain variable region (IgVH) mutational status, CD38 expression, chromosomal aberrations (del17p, del11q, del13q, and Tri12), and Rai staging were performed as previously described [47]. PBMCs were separated by density centrifugation using Biocoll (Biochrom AG). CLL cells were isolated by untouched magnetic cell sorting (EasySep, StemCell Tech), and purity was assessed by CD5CD19 staining using flow cytometry.

### Flow cytometry and cell sorting

For flow cytometry, the following antibodies were used: Polyclonal Rabbit FITC anti-human IgG F(ab′)2 (DAKO), PE anti-human CD86 and isotype control (Biolegend), Alexa Fluor 488 anti-human CD86 and isotype control (Biolegend), Alexa Fluor 647 anti-human CD19 (ebioscience), PE anti-human CD184 and isotype control (BD-Pharmingen), allophycocyanin-Alexa Fluor 700 anti-human CD5 (Beckman Coulter), FITC anti-human CD5 (Beckman Coulter), FITC anti-human Ki67 and isotype control (BD-Pharmigen). For intracellular Ki67 staining, cells were stained for CD5, CD19, CD86 prior to fixation and permeabilization using the Cytofix/Cytoperm Fixation/Permeabilization Kit (BD-Pharmigen). For cell cycle analysis, cells were stained with antibodies for CD5, CD19, and CD86 and washed twice with ice cold PBS prior to fixation in 80% EtOH for 30 min. Cells were washed twice in ice cold PBS and incubated in 1 μg/mL DAPI 0.1% Triton X-100 solution for 30 min and immediately analyzed by flow cytometry. Cells were analyzed on a Gallios Flow Cytometer (Beckman Coulter). For sorting of CD86^+^ CLL cells, cells were stained using CD5-FITC and CD86-PE antibodies and CD86 positive cells were purified on an Epics Altra Cell Sorter (Beckman Coulter).

### Fluorescence microscopy

Cell culture slides (BD Falcon) were coated with 0.5 mL 0.01% poly-l-lysine solution (Sigma) for 1 h at RT and washed with sterile water. A total of 5 × 10^6^ CLL cells were stained with fixable viability dye (ebioscience) to exclude apoptotic cells, resuspended in 1 mL RPMI complete, transferred to coated cell culture slides and incubated for 1 h at 37°C. Cells were washed three times with PBS, followed by fixation with 500 μL 4% paraformaldehyde (Sigma) in PBS for 15 min. Fixed cells were washed with PBS, twice with TBS, and subsequently permeabilized with 0.2% saponine (Sigma)/1% HSA (Calbiochem) in TBS for 5 min. Three washing steps with washing buffer (0.05% saponine/1% HSA in TBS) were followed by blocking with 250 μL blocking solution (5% donkey serum (Jackson immunoresearch) in washing buffer). Cells were washed three times with washing buffer and stained using PE anti-human CD19 (BD-Pharmingen), Alexa Fluor 488 anti-human CD86 (Biolegend), and Alexa Fluor 647 conjugated anti-γ-H2AX antibodies (Biolegend) overnight at 4°C in a wet chamber. Stained cells were washed three times with washing buffer, two times with sterile water, and mounted in ProLong Antifade reagent (Invitrogen). Slides were analyzed by fluorescence microscopy at ambient temperature (Olympus IX81 fluorescence microscope; 60x oil using PLAPON60XO/1,42 objective lenses with 1.42 numerical aperture; DP72 CCD camera, OLYMPUS Xcellence System acquisition and processing software; linear brightness and contrast adjustment was performed to distinguish CD86+ and CD86− cells and to distinguish γ-H2AX foci from background staining).

### IgV and Sμ mutations

RNA and genomic DNA was isolated from highly purified CLL samples using High Pure PCR Template Preparation Kit and High Pure RNA Isolation Kit (Roche). cDNA was generated from RNA using reverse transcription (iScript, Bio-Rad). Rearranged IgV regions were PCR amplified from cDNA according to [48] using V-specific forward primers (vh1/vh7: 5′-atggactggacctggagg-3′, vh2: 5′-cacrctcctgctgctgac ca-3′, vh3 a: 5′-gct ggg ttt tcc ttg ttg c-3′, vh3b: 5′-atg gag ttr ggr ctg agc tg-3′, vh4: 5′-gctcccagatggggtcctg-3′, vh5: 5′-ctcctcctggctgttctcc-3′, vh6: 5′-ctgtctccttcctcatcttcc-3′) and IgM-specific revers primer (5′-CAGGAGAAAGTGATGGAGTCG-3′). DNA sequences from amplified VDJ regions were determined by Sanger sequencing (MWG Eurofins; Germany) and the respective VDJ regions were identified using IMGT/V-quest search page at www.IMGT.org [49],[50]. After determining the VDJ usage of the samples, the genomic region spanning VDJ to Sμ was PCR amplified (Phusion proof-reading Polymerase, Biozym), using an Sμ-specific reverse primer in combination with V-specific forward primers (primer list in Supporting Information Table 2). The gel-purified (Qiagen) PCR product was subjected to second round PCR using tagged primers (listed in Supporting Information Table 2). The PCR products were gel purified (Qiagen) and pooled. The pooled PCR products were paired-end sequenced on the Miseq platform and forward and reverse reads were merged (Illumina; GATC-Biotech, Germany). The amplicon sequences corresponding to one PCR reaction were compared with each other. The most common sequence was determined. The remaining sequences were ordered with regards to their abundances and numbers of mutations with respect to the most common one. Positions, types, and associated Phred scores of all mutations were recorded. The sequences were then filtered according to the following rule: for each mutation, the average Phred score across all sequences that had that mutation (as given by position and type of nucleotide exchange) was calculated. If the resulting average Phred score had a value ≤20, the sequence was excluded. If a sequence had more than one mutation, all mutations had to pass above threshold. Custom R and Perl scripts were used at all steps. Genealogical relations and phylogenetic trees were generated using the phylogenie.fr online tool [51].

### Statistics

Statistical analysis was performed with IBM SPSS 20 and GraphPad Prism 5. Mann–Whitney test (nonnormally distributed samples), paired Student's *t*-tests (normally distributed samples), and the Log-Rank test for Kaplan–Meier analysis were used to determine significance.

## Abbreviations

AID: activation-induced deaminase
CLL: chronic lymphocytic leukemia
ds: double strand
IgV: immunoglobulin variable
IgV-UM: immunoglobulin variable domain unmutated
IgV-Mut: immunoglobulin variable domain mutated
NGS: next generation sequencing
Sμ: IgM switch region

## Supporting Information

As a service to our authors and readers, this journal provides supporting information supplied by the authors. Such materials are peer reviewed and may be re-organized for online delivery, but are not copy-edited or typeset. Technical support issues arising from supporting information (other than missing files) should be addressed to the authors.

Fig. S1. Dominant VDJ sequences of IgV-UM #1, 2 and IgV-Mut #3, 4 including all subclonal variations. Fig. S2. Stability of CD86 expression. CD86 levels were measured from 11 freshly prepared CLL samples. Aliquots from each sample were frozen at liquid nitrogen and reanalyzed again upon thawing. Table S1. patient details Table S2. Primer list

Table S3. Sequences derived from deep resequencing of VDJ and Sμ regions

Table S4. Comparison of sequences in unsorted and CD86^+^ sorted cells with a frequency >0.1%
