# Supplementary material for: AID induces intraclonal diversity and genomic damage in CD86+ chronic lymphocytic leukemia cells
Source: Eur J Immunol. 2014 Oct 18;44(12):3747–57. doi: 10.1002/eji.201344421 (PMC4276288; doi:10.1002/eji.201344421)
Supplement: Supplementary file 1 — Fig. S1. Dominant VDJ sequences of IgV-UM #1, 2 and IgV-Mut #3, 4 including all subclonal variations. Fig. S2. Stability of CD86 expression. CD86 levels were measured from 11 freshly prepared CLL samples. Aliquots from each sample were frozen at liquid nitrogen and reanalyzed again upon thawing. Table S1. patient details Table S2. Primer list [file eji0044-3747-sd1.pdf]

European Journal of Immunology

Supporting Information for DOI 10.1002/eji.201344421

Michael Huemer, Stefan Rebhandl, Nadja Zaborsky, Franz J. Gassner, Stefan Hainzl, Lukas Weiss, Daniel Hebenstreit, Richard Greil and Roland Geisberger

AID induces intraclonal diversity and genomic damage in CD86^{+} chronic lymphocytic leukemia cells

© 2014 WILEY-VCH Verlag GmbH & Co. KGaA, Weinheim

Supporting information

Supporting information Fig. 1. Dominant VDJ sequences of IgV-UM #1, 2 and IgV-Mut #3, 4 including all subclonal variations.

Supporting information Fig. 2. Stability of CD86 expression. CD86 levels were measured from 11 freshly prepared CLL samples. Aliquots from each sample were frozen at liquid nitrogen and reanalyzed again upon thawing.

Supporting information Table 1. patient details

Supporting Information Table 2. Primer list

Supporting information Table 3. Sequences derived from deep re-sequencing of VDJ and Sμ regions

Supporting information Table 4. Comparison of sequences in unsorted and $CD86^+$ sorted cells with a frequency >0.1%

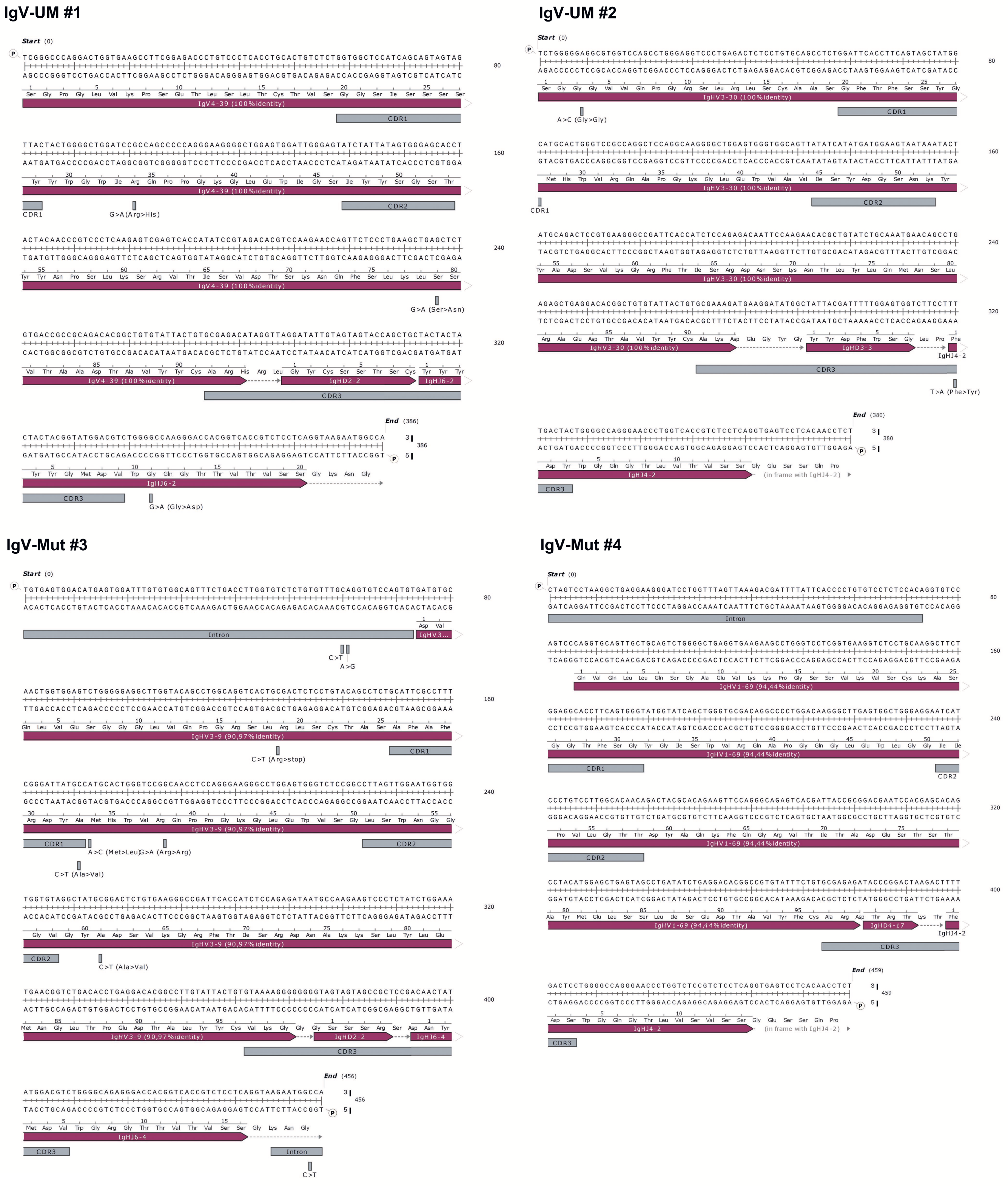

Supporting information Fig. 1. Dominant VDJ sequences of IgV-UM #1, 2 and IgV-Mut #3, 4 including all subclonal variations.

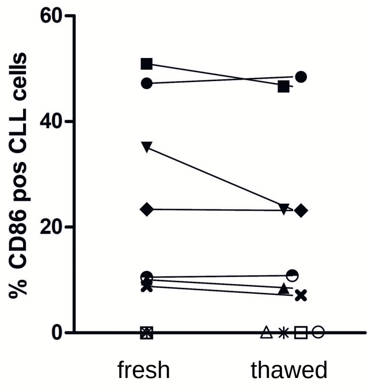

Supporting Information Fig. 2. Stability of CD86 expression. CD86 levels were measured from 11 freshly prepared CLL samples. Aliquots from each sample were frozen at liquid nitrogen and reanalyzed again upon thawing.

Supporting Information Table 1. Patient details

The following 4 patient samples were subjected to deep sequencing (shown in Fig 1)

	Patient ID	Sex	age at diagnosis (years)	Time between diagnosis and sampling (months)	treatment	Rai stage	Mut stat	Zytogen	Zap70	CD38	VLA4
IgV-UM #1	149	M	68,00	9,00	untreated	2	UM	Tri12	↑	↑	↑
IgV-UM #2	337	M	65,00	51,00	treated	1	UM	del11q 13q	↓	↑	↓
IgV-Mut #3	154	M	69,00	62,00	treated	2	M	del13q	↓	↓	n.d.
IgV-Mut #4	283	M	57,00	52,00	treated	2	M	del11q	↑	↓	↓

Supplementary table T1 (patient details)

patients included in time to first treatment statistics (Fig 5 and table 1)
n.d.=not determined

Patient ID	Sex	age at diagnosis (years)	Time between diagnosis and sampling (months)	treatment	time to first treatment (months)	Rai stage	Mut stat	Zytogen	Zap70	CD38	VLA	CD86+ (%)
6	F	52,00	36,00	treated	43,4	2	M	del11q Tri12	↓	↓	↑	4,27
16	F	74,00	55,00	treated	77,0	1	UM	Tri12	↑	↑	↑	0,89
40	M	64,00	126,00	treated	138,0	1	M	del13q	↓	↓	n.d.	4,85
72	M	76,00	140,00	untreated	152,6	0	n.d.	del13q	↓	↓	↓	1,12
85	M	53,00	44,00	treated	67,9	2	M	del13q	↓	↓	↑	1,88
106	F	75,00	89,00	untreated	102,6	2	M	normal	n.d.	↓	↓	1,66
109	F	70,00	189,00	untreated	202,6	1	M	Tri12	↓	↓	↑	0,46
116	F	60,00	95,00	untreated	104,6	1	M	del13q	↓	↓	n.d.	1,61
123	M	63,00	115,00	untreated	128,6	2	M	del13q	↓	↓	↓	0,09
137	F	69,00	142,00	untreated	154,6	0	UM	Tri12	↑	↑	↑	0,42
149	M	68,00	9,00	treated	53,8	2	UM	Tri12	↑	↑	↑	3,91
151	M	74,00	81,00	untreated	92,6	1	M	del13q	↓	↓	↓	1,3
154	M	69,00	62,00	treated	63,7	2	M	del13q	↓	↓	n.d.	7,36
155	M	57,00	24,00	treated	25,0	2	n.d.	normal	n.d.	↓	n.d.	11,9
157	F	59,00	83,00	untreated	94,5	1	M	del13q	↓	↓	↓	15,32
158	F	68,00	118,00	untreated	130,7	2	M	del13q	↓	↓	↓	2,45
181	M	72,00	23,00	treated	70,1	1	UM	Tri12	↑	↓	↑	0,95
230	F	70,00	9,00	treated	66,8	0	UM	del13q	↑	↑	↑	1,16
232	F	79,00	68,00	untreated	81,6	0	M	del13q 17p	n.d.	↓	n.d.	1,4
242	F	56,00	69,00	untreated	79,6	1	UM	del13q	↑	↓	↓	0,59
264	M	69,00	87,00	untreated	96,7	0	M	del13q	↑	↑	↑	0,01
279	M	57,00	63,00	untreated	75,0	1	M	normal	↓	↓	↑	7,51
280	F	74,00	62,00	untreated	73,8	0	M	normal	n.d.	↓	↑	1,28
283	M	57,00	52,00	treated	51,5	2	M	del11q	↑	↓	↓	3,01
289	M	59,00	53,00	untreated	61,6	0	UM	normal	↑	↑	n.d.	0,25
305	F	65,00	15,00	treated	16,0	1	n.d.	normal	↑	↑	n.d.	15,7
309	M	75,00	10,00	treated	18,5	3	UM	del11q	n.d.	↓	n.d.	6,35
319	F	56,00	69,00	untreated	82,7	0	M	del13q	↓	↓	↓	2,98
321	M	62,00	45,00	treated	44,7	4	UM	del13q 17p Tri12	↑	↑	↑	1,27
326	F	81,00	3,00	treated	22,0	3	UM	normal	↓	↓	n.d.	0,86
353	M	63,00	99,00	treated	123,6	2	M/UM	normal	↑	↑	↑	2,96
359	M	50,00	47,00	untreated	61,6	0	n.d.	del13q	↓	↓	↑	0,97
366	M	66,00	43,00	treated	70,3	2	M	del11q	↓	↓	↓	0,53
386	M	46,00	48,00	untreated	56,6	0	M	normal	↓	↓	↓	0,91
399	F	76,00	60,00	untreated	74,3	0	M	normal	↓	↑	↓	0,56
411	F	72,00	48,00	treated	60,3	0	UM	del13q	n.d.	↓	↑	0,55
419	M	69,00	38,00	untreated	51,6	1	M	del13q	↓	↓	↓	0,45
422	F	43,00	34,00	untreated	70,7	0	M	normal	↑	↓	↓	17,97
435	F	60,00	7,00	treated	43,6	2	M	del13q	↓	↓	↓	12,8
442	M	61,00	50,00	untreated	59,6	0	M	del13q	↓	↑	n.d.	0,03
447	M	68,00	56,00	untreated	67,6	0	M	del13q	↓	↓	↓	0,18
453	M	66,00	39,00	untreated	49,2	1	n.d.	del11q 13q	↑	↑	↓	1,19
456	M	66,00	9,00	treated	20,8	1	UM	del11q	↑	↑	↓	0,08
485	M	60,00	123,00	untreated	130,7	0	n.d.	del13q	↓	↓	↓	1,37
489	M	81,00	36,00	untreated	49,6	0	n.d.	del11q 13q	↑	↓	↓	5,3
496	M	69,00	31,00	untreated	44,6	1	M	normal	↓	↓	↓	1,72
501	F	65,00	3,00	treated	3,3	0	UM	normal	↑	↑	n.d.	1,54
503	M	47,00	60,00	untreated	67,6	0	n.d.	del11q 13q	↑	↓	n.d.	0,04
518	F	77,00	23,00	untreated	35,6	0	n.d.	normal	↑	↑	n.d.	0,48
530	M	80,00	7,00	treated	21,3	0	n.d.	del11q 13q	↑	↑	↓	3,51
537	F	67,00	122,00	untreated	130,7	n.d.	UM	del13q	↓	↓	n.d.	0,37
560	M	66,00	8,00	untreated	20,6	1	n.d.	del13q	↓	↓	n.d.	2,68
564	F	72,00	88,00	untreated	100,6	n.d.	n.d.	n.d.	n.d.	n.d.	n.d.	4,29
565	M	60,00	12,00	untreated	23,7	0	n.d.	del13q	n.d.	n.d.	n.d.	0,17
577	M	60,00	86,00	treated	93,6	n.d.	M	del13q	↑	↑	↑	1,91
578	F	59,00	24,00	untreated	31,9	0	n.d.	del13q	↓	↓	n.d.	0,64
582	F	68,00	17,00	untreated	24,7	1	n.d.	normal	↓	↓	n.d.	0,15
587	M	74,00	46,00	untreated	57,6	0	n.d.	n.d.	n.d.	n.d.	n.d.	0,5
594	F	61,00	8,00	treated	8,6	1	n.d.	n.d.	↑	↑	↓	0,21
median		66,0	48,0		66,8							1,3
±SD		8,7	41,0		40,6							4,1

patients included in CXCR4 CD86 statistics (Fig 2)
n.d.=not determined

Patient ID	Mut status	Zytogenetics	Zap70	CD38	VLA4	Therapy
2	M	del13q	↓	↓	↓	treated
14	M	del13q	↓	↓	↑	treated
23	n.d.	del17p	n.d.	↑	↓	treated
45	M	del13q	↓	↓	↓	treated
60	M	del13q	↓	↓	↓	treated
61	M	del13q	↓	↓	↓	treated
72	n.d.	del13q	↓	↓	↓	untreated
75	M	del13q	↓	↓	↓	treated
106	M	normal	n.d.	↓	↓	untreated
108	M	del13q	↓	↓	↓	untreated
109	M	Tri12	↓	↓	↑	untreated
116	M	del13q	↓	↓	n.d.	untreated
123	M	del13q	↓	↓	↓	untreated
137	UM	Tri12	↑	↑	↑	untreated
158	M	del13q	↓	↓	↓	untreated
200	UM	del13q	↑	↑	↑	treated
232	M	del13q 17p	n.d.	↓	n.d.	untreated
240	UM	normal	↑	↓	n.d.	treated
279	M	normal	↓	↓	↑	untreated
283	M	del11q	↑	↓	↓	treated
308	M	normal	↓	↓	↓	treated
319	M	del13q	↓	↓	↓	untreated
321	UM	del13q 17p Tri12	↑	↑	↑	treated
359	n.d.	del13q	↓	↓	↑	untreated
366	M	del11q	↓	↓	↓	treated
386	M	normal	↓	↓	↓	untreated
399	M	normal	↓	↑	↓	untreated
419	M	del13q	↓	↓	↓	untreated
422	M	normal	↑	↓	↓	untreated
435	M	del13q	↓	↓	↓	untreated
447	M	del13q	↓	↓	↓	untreated
453	n.d.	del11q 13q	↑	↑	↓	untreated
456	UM	del11q	↑	↑	↓	treated
467	UM	del13q 17p	↑	↑	n.d.	treated
485	n.d.	del13q	↓	↓	↓	untreated
489	n.d.	del11q 13q	↓	↓	↓	untreated
496	M	normal	↓	↓	↓	untreated
503	n.d.	del11q 13q	↑	↓	n.d.	untreated
518	n.d.	normal	↑	↑	n.d.	untreated
530	n.d.	del11q 13q	↑	↑	↓	treated
531	n.d.	del13q	↑	↓	↓	untreated
537	UM	del13q	↓	↓	n.d.	untreated
560	n.d.	del13q	↓	↓	n.d.	untreated
564	n.d.	n.d.	n.d.	n.d.	n.d.	untreated
565	n.d.	del13q	n.d.	n.d.	n.d.	untreated
577	n.d.	del13q	↑	↑	n.d.	untreated
578	n.d.	del13q	↓	↓	n.d.	untreated
582	n.d.	normal	↓	↓	n.d.	untreated
587	n.d.	n.d.	n.d.	n.d.	n.d.	untreated
594	n.d.	n.d.	↑	↑	n.d.	treated

patients included in Ki67 CD86 statistics (Fig 2)
n.d.=not determined

Patient ID	Mut status	Zytogenetics	Zap70	CD38	VLA4	Therapy
2	M	del13q	↓	↓	↓	treated
23	n.d.	del17p	n.d.	↑	↓	treated
45	M	del13q	↓	↓	↓	treated
61	M	del13q	↓	↓	↓	treated
72	n.d.	del13q	↓	↓	↓	untreated
116	M	del13q	↓	↓	n.d.	untreated
151	M	del13q	↓	↓	↓	untreated
187	UM	del13q	↓	↑	↑	treated
200	UM	del13q	↑	↑	↑	treated
240	UM	normal	↑	↓	n.d.	treated
264	M	del13q	↑	↑	↑	untreated
279	M	normal	↓	↓	↑	untreated
283	M	del11q	↑	↓	↓	treated
289	M	normal	↓	↓	↓	untreated
293	UM	normal	n.d.	↑	↓	untreated
321	UM	del13q 17p Tri12	↑	↑	↑	treated
359	n.d.	del13q	↓	↓	↑	untreated
366	M	del11q	↓	↓	↓	treated
386	M	normal	↓	↓	↓	untreated
422	M	normal	↑	↓	↓	untreated
442	M	del13q	↓	↑	n.d.	untreated
447	M	del13q	↓	↓	↓	untreated
453	n.d.	del11q 13q	↑	↑	↓	untreated
456	UM	del11q	↑	↑	↓	treated
467	UM	del13q 17p	↑	↑	n.d.	treated
485	n.d.	del13q	↓	↓	↓	untreated
489	n.d.	del11q 13q	↓	↓	↓	untreated
496	M	normal	↓	↓	↓	untreated
503	n.d.	del11q 13q	↑	↓	n.d.	untreated
531	n.d.	del13q	↑	↓	↓	untreated
537	UM	del13q	↓	↓	n.d.	untreated
564	n.d.	n.d.	n.d.	n.d.	n.d.	untreated
565	n.d.	del13q	n.d.	n.d.	n.d.	untreated
577	n.d.	del13q	↑	↑	n.d.	untreated
578	n.d.	del13q	↓	↓	n.d.	untreated
582	n.d.	normal	↓	↓	n.d.	untreated
587	n.d.	n.d.	n.d.	n.d.	n.d.	untreated
594	n.d.	n.d.	↑	↑	n.d.	treated

patient samples used for H2AX staining (Fig 4)
n.d.=not determined

Patient ID	Sex	age at diagnosis (years)	Time between diagnosis and sampling (years)	treatment	Rai stage	Mut stat	Zytogen	Zap70	CD38	VLA-4
210	F	57	11.48	untreated	0	M	Del13q	↓	↓	↓
36	F	47	15.42	2001: Fludarabine	2	n.d.	n.d.	↓	↑	
489	M	81	4.67	untreated	n.d.	M	Del11q 13q	↓	↓	↓
338	M	63	5.93	untreated	0	M	Del13q	↓	↓	↓

patient samples used for DAPI analysis (Fig 2)
n.d.=not determined

Patient ID	Sex	age at diagnosis (years)	Time between diagnosis and sampling	treatment	Rai stage	Mut stat	Zytogen	Zap70	CD38	VLA-4
472	F	72	57	untreated	0	M	del13q	↓	↓	↑
565	M	60	21	untreated	0	UM	del13q	↑	↑	↓
13	M	76	111	untreated	1	M	del13q 17p	↓	↓	↓
262	M	62	83	untreated	1	M	del13q	↓	↓	↑
417	M	79	65	untreated	0	n.d.	del13q	↑	↑	↓

Supporting Information Table 2. Primer list

ID Fig. 1	Patient ID	Lab-internal sample ID	primer 1st round	sequence 5'-3'	amplified region	primer 2nd round	sequence 5'-3'	amplified region
IgV-UM #1	ID: 149	3157	fwd RG686	gtcctgtcccagctgcagctgcaggag	V-Sμ	fwd RG698	AATCGCgtcctgtcccagctgcagctgcaggag	VDJ
						rev RG699	AATCGCAGAAAACAAAGGCCCTAGAG	
			rev Spl9	AAA AAT GAG CAG GTC CCC TT		fwd RG700	AATCGCGTATTTGACGTCTTATTTCCACTAGAAGG	Sμ
						rev RG701	AATCGCAAAAATGAGCAGGTCCCCTT	
IgV-UM #2	ID: 337	7805	fwd RG687	tgtccagtgtcaggtgcagctggtggag	V-Sμ	fwd RG702	TGAGCAtgtccagtgtcaggtgcagctggtggag	VDJ
						rev RG703	TGAGCAACCCTTCAGAGTTAAAGCAGGAG	
			rev Spl9	AAA AAT GAG CAG GTC CCC TT		fwd RG704	TGAGCAGTATTTGACGTCTTATTTCCACTAGAAGG	Sμ
						rev RG705	TGAGCAAAAATGAGCAGGTCCCCTT	
IgV-Mut #3	ID: 154	4663	fwd RG691	ggtgattcatggagaaatagagagattgag	V-Sμ	fwd RG706	GTCTAGgtgattcatggagaaatagagagattgag	VDJ
						rev RG707	GTCTAGCAGAAAACAAAGGCCCTAGAG	
			rev Spl9	AAA AAT GAG CAG GTC CCC TT		fwd RG708	GTCTAGGTATTTGACGTCTTATTTCCACTAGAAGG	Sμ
						rev RG709	gtctagAAAAATGAGCAGGTCCCCTT	
		4663 CD86 sorted	fwd RG691	ggtgattcatggagaaatagagagattgag	V-Sμ	fwd RG714	agtcctggtgattcatggagaaatagagagattgag	VDJ
			rev Spl9	AAA AAT GAG CAG GTC CCC TT		rev RG715	agtcctGCAGAAAACAAAGGCCCTAGAG	
IgV-Mut #4	ID: 283	4788	fwd RG688	CAGCAGCTACAggtaaggggcttc	V-Sμ	fwd RG718	taagacAGCAGCTACAggtaaggggcttc	VDJ
						rev RG719	taagacAACCCTTCAGAGTTAAAGCAGGAG	
			rev Spl9	AAA AAT GAG CAG GTC CCC TT		fwd RG720	taagacGTATTTGACGTCTTATTTCCACTAGAAGG	Sμ
						rev RG721	taagacAAAAATGAGCAGGTCCCCTT	
		4788 CD86 sorted	fwd RG688	CAGCAGCTACAggtaaggggcttc	V-Sμ	fwd RG726	ctgaggCAGCAGCTACAggtaaggggcttc	VDJ
			rev Spl9	AAA AAT GAG CAG GTC CCC TT		rev RG727	ctgaggAACCCTTCAGAGTTAAAGCAGGAG	
